# NSF Workshop Report: Exploring Measurements and Interpretations of Intelligent Behaviors Across Animal Model Systems

**DOI:** 10.1002/cne.70035

**Published:** 2025-03-04

**Authors:** Joseph V. Gogola, Mary Kate Joyce, Susheel Vijayraghavan, George Barnum, Gregg Wildenberg

**Affiliations:** ^1^ Department of Medicine The University of Chicago Chicago Illinois USA; ^2^ Department of Neuroscience Yale University School of Medicine New Haven Connecticut USA; ^3^ Department of Physiology and Pharmacology Schulich School of Medicine and Dentistry, Western University London Ontario Canada; ^4^ Department of Computation and Neural Systems California Institute of Technology Pasadena California USA; ^5^ Department of Neurobiology The University of Chicago Chicago Illinois USA

## Abstract

Defining intelligence is a challenging and fraught task, but one that neuroscientists are repeatedly confronted with. A central goal of neuroscience is to understand how phenomena like intelligent behaviors emerge from nervous systems. This requires some determination of what defines intelligence and how to measure it. The challenge is multifaceted. For instance, as we begin to describe and understand the brain in increasingly specific physical terms (e.g., anatomy, cell types, activity patterns), we amplify an ever‐growing divide in how we connect measurable properties of the brain to less tangible concepts like intelligence. As our appreciation for evolutionary diversity in neuroscience grows, we are further confronted with whether there can be a unifying theory of intelligence. The National Science Foundation (NSF) NeuroNex consortium recently gathered experts from multiple animal model systems to discuss intelligence across species. We summarize here the different perspectives offered by the consortium, with the goal of promoting thought and debate of this ancient question from a modern perspective, and asking whether defining intelligence is a useful exercise in neuroscience or an ill‐posed and distracting question. We present data from the vantage points of humans, macaques, ferrets, crows, octopuses, bees, and flies, highlighting some of the noteworthy capabilities of each species within the context of each species’ ecological niche and how these may be challenged by climate change. We also include a remarkable example of convergent evolution between primates and crows in the circuit and molecular basis for working memory in these highly divergent animal species.

## Graphical abstract

• Overview of challenges in defining and measuring intelligence in neuroscience, considering evolutionary diversity and ecological niches.

• Insights from humans, macaques, ferrets, crows, octopuses, bees, and flies, including how intelligence manifests in their respective contexts.

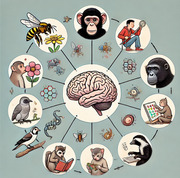

## 1 IQ or Not IQ: That is the Question

Defining intelligence is one of the most central challenges in neuroscience, yet it still does not have an agreed‐upon singular definition. Traditional approaches to intelligence have largely centered on human cognition, creating a narrow and potentially biased understanding of this complex phenomenon (Bräuer et al. 2020; Katz 2019). This human‐centric focus has been highly informative but, by definition, has hindered a more comprehensive understanding of intelligence broadly. A broader perspective is essential to uncover the underlying principles and mechanisms that drive intelligent behavior.

A key problem in defining intelligence more broadly is that research has become more anthropocentric, perhaps driven by the push toward more translational science (Zerhouni 2003; Zerhouni 2005). This has led to a decreasing breadth of model species for research inquiry (Farris 2020; Laurent 2020). The problem is further compounded by the availability of modern tools for only a small number of species (Irion and Nusslein‐Volhard 2022; Davis 2004). As a result, studies of intelligence have suffered perhaps the biggest hit in the generalizability of findings.

While the focused approach has yielded valuable insights into intelligence, it has also limited our ability to appreciate the diversity of intelligence across the animal kingdom. Thus, there is a need to return to using a wider phylogenetic lens in examining intelligence. To address this, recent efforts have called for a more comparative approach (Laurent 2020), exploring how different species solve problems and adapt to their environments, both hallmarks of intelligence. By examining a wide range of organisms, from insects to primates, we can gain a more comprehensive picture of intelligence and potentially identify core cognitive processes that are shared across species.

These goals were the foundation of the 2023 NeuroNex Annual Meeting, hosted by the National Science Foundation, “From Octopus to Macaque: The Wondrous Challenges of Diverse Nervous Systems.” The meeting brought together scientists from a wide swath of fields, interests, and model systems to attempt to broach these questions. The goal of this meeting was to explore diverse types of intelligence across species, including the remarkable behaviors of octopuses, crows, and primates, and how differences in nervous system structure might help elucidate functional mechanisms that mediate intelligence. In addition, a key focus of the meeting was on modern technologies for neurobiological inquiry, as well as the challenges faced in adapting and utilizing these technologies in non‐rodent species. What follows is not an attempt at answering those questions, nor definitive statements on the benchmarks of intelligence, and when and how they should be applied. Instead, we present key highlights from this meeting, primarily on work done by its speakers in the context of broader neuroscientific inquiry, to form the bedrock of how to deliberate on a key question in neuroscience: what defines intelligence?

## 2 Human Error: The Limits of Anthropocentric Definitions of Intelligence

### 2.1 What Operations Are Valued for Intelligence?

Notably, there is a lack of consensus on the definition of intelligence (Legg and Hutter 2007). We provide a few examples from a variety of definitions, many of which center around goal‐directed and flexible behavior, particularly in changing environments (Table 1). We do not promote one definition over another but offer these as a conceptual bedrock in thinking about how intelligence has been described historically and how it might be described moving forward. For the sake of coherence, we will consider intelligence in the framework offered by Legg and Hutter (2007) as a common, informal definition—*“Intelligence measures an agent's ability to achieve goals in a wide range of environments*.”

**TABLE 1:** Some definitions of intelligence, non‐exhaustive.

Type	Quote	Source
Collectives	“Intelligence is a very general mental capability that, among other things, involves the ability to reason, plan, solve problems, think abstractly, comprehend complex ideas, learn quickly and learn from experience.”	Common statement with 52 expert signatories
	“Individuals differ from one another in their ability to understand complex ideas, to adapt effectively to the environment, to learn from experience, to engage in various forms of reasoning, to overcome obstacles by taking thought.”	American Psychological Association
Psychologists	“Intelligence is assimilation to the extent that it incorporates all the given data of experience within its framework… There can be no doubt either that mental life is also accommodation to the environment. Assimilation can never be pure because by incorporating new elements into its earlier schemata, the intelligence constantly modifies the latter in order to adjust them to new elements.”	J. Piaget
	“Ability to adapt oneself adequately to relatively new situations in life.”	R. Pinter
	“… adjustment or adaptation of the individual to his total environment, or limited aspects thereof… the capacity to reorganize one's behavior patterns so as to act more effectively and more appropriately in novel situations… the ability to learn… the ability to carry on abstract thinking… the effective use of concepts and symbols in dealing with a problem to be solved… ”	W. Freeman
AI Researchers	“Any system… that generates adaptive behaviour to meet goals in a range of environments can be said to be intelligent.”	D. Fogel
	“… doing well at a broad range of tasks is an empirical definition of ‘intelligence.’”	H. Masum
	“Achieving complex goals in complex environments”	B. Goertzel
A Common Definition (Legg and Hutter 2007)	“Intelligence measures an agent's ability to achieve goals in a wide range of environments.”	Legg and Hutter (2007)

*Note:* We provide some definitions of intelligence from a variety of primary sources, initially compiled by Legg and Hutter (2007). Notably, they offer at least 71 separate definitions of intelligence, of which we have chosen a select few. We also provide their attempt at condensing a variety of features into a single definition of intelligence.

Describing intelligence often begins with an anthropocentric perspective, where intelligence is viewed through the lens of human achievements and capabilities (Arjonilla and Kobayashi 2019). In humans, intelligence encompasses a wide array of mental abilities that have almost certainly allowed our species to thrive across diverse habitats. These abilities span a wide array of domains. For instance, human intelligence is marked by the capacity for future planning, such as creating detailed strategies to achieve long‐term goals (Sternberg 2019), which has allowed us to exploit and survive in various habitats. Another key element of human intelligence is social organization, facilitated by language, which enables complex communication and cooperation (Premack 2004). This social aspect of intelligence is seen in the formation of alliances, hierarchical structures, and collective problem‐solving. Abstract thinking, another hallmark of human intelligence, allows for the development of theories, art, and philosophy, further distinguishing human cognitive capabilities (Dennett 1994). Based on the enlargement of the prefrontal cortex in humans, which is known to underlie abstract thinking, flexible language production, and planning for the future, it is presumed that these functions are especially developed in humans (Levy 2024).

While this human‐centric view provides a framework for understanding intelligence, it is essential to recognize that animal intelligence cannot be reduced to these human‐specific traits. To some first approximation, not all human traits we might define as intelligent have direct equivalencies in nonhuman animals. Nonhuman animals may not exhibit the same forms of intelligence as humans, but they possess cognitive abilities that are highly adapted to their ecological niches (Irion and Nusslein‐Volhard 2022). For example, the ability to navigate, find food, and avoid predators are crucial in shaping animal intelligence and may not require the same level of abstract thinking or language‐based communication seen in humans. Yet, humans are also animals and undoubtedly faced similar ecological pressures as other animals in the evolutionary past. Therefore, it is possible, if not probable, that intelligent behaviors might be borne out of overarchingly similar neural bases. Thus, further development in the experimental tools and techniques by which we can probe animals on these more abstract or conceptual thinking, as well as a greater focus on ethologically relevant paradigms with which to probe these cognitive abilities across various animals, are essential to delineating these potential similarities.

### 2.2 Schema Cells and Persistent Activity: Neural Bases Underlying Cognitive Maps and Working Memory in Macaques

In studying the neural basis of intelligence, researchers often turn to nonhuman primates, such as macaques, due to their cognitive similarities to humans (Gray and Barnes 2019). One key area of research is the role of schema cells in the macaque hippocampus, as highlighted by Sylvia Wirth et al. Schema cells are crucial for organizing and retrieving memory, forming cognitive maps and schemas that enable macaques to navigate and plan within their environments. Their recent work has demonstrated that the hippocampus encodes recurring patterns across different environments, establishing cognitive schemas akin to semantic information in humans (Baraduc et al. 2019). This ability to form and utilize schemas allows macaques to generalize knowledge and apply it to new situations, facilitating adaptive behavior and decision‐making in their dynamic environments.

Another significant aspect of the neural basis of intelligence in macaques is working memory, which Andreas Nieder has extensively studied (Nieder 2022). Working memory is essential for higher cognitive functions such as reasoning, problem‐solving, and planning. Nieder and Wirth's research has shown that macaques can hold and manipulate information in working memory to perform complex tasks, such as making inferences about social hierarchies or planning routes in virtual environments. This ability to maintain and work with information over short periods underpins many aspects of macaque cognition, enabling them to adapt to new challenges and solve problems in real‐time. The prefrontal cortex, a brain region associated with working memory, plays a critical role in these cognitive processes, supporting the flexible and goal‐directed behavior observed in macaques (Jacob and Nieder 2014).

Beyond schema cells and working memory, macaques excel in various cognitive tasks that highlight the complexity of their neural architecture. For instance, they demonstrate remarkable capabilities in relational learning, identifying similarities, and mapping functional equivalences between different environments. They can navigate virtual environments, plan trajectories to reach goals, and understand the functional equivalences between different settings (Wirth et al. 2017). This ability to recognize and utilize relational patterns is supported by a network of brain regions, including the prefrontal cortex and hippocampus. These regions work together to encode and retrieve information, allowing macaques to perform complex cognitive tasks that require integrating multiple sources of information. By studying these neural bases, researchers gain insights into the fundamental mechanisms of intelligence that are shared across species, providing a deeper understanding of how cognitive abilities evolve and function in diverse ecological contexts.

### 2.3 Flexible Representations Enable Highly Social Species to Survive a Complex, Ever‐Changing Environment

Flexible representations are vital for survival, especially in highly social species like humans and macaques that inhabit complex and ever‐changing environments. These representations allow individuals to adapt to changing conditions, form social bonds, and innovate and solve problems.

In macaques, flexible representations are crucial for navigating dynamic social hierarchies and environmental conditions. Macaque society, which shares many features with human society, such as dominance hierarchies and alliances, requires sophisticated cognitive abilities to maintain and understand complex social relationships. This includes recognizing individuals, understanding social hierarchies, and predicting others’ behavior. Research has shown that macaques possess a multimodal representation that binds the face and voice identity of others, which is essential for maintaining these social relationships. For example, neurons in the orbitofrontal cortex represent social categories such as age, gender, and emotions, which are critical for environmental navigation (Barat 2018). This ability to integrate and use social information highlights the importance of flexible representations in macaque cognition.

In addition to social cognition, macaques demonstrate flexibility in spatial and relational representations. Studies by Sylvia Wirth et al. have shown that macaques can navigate virtual environments, plan trajectories to reach goals, and understand the functional equivalences between different settings. This ability to recognize and utilize relational patterns is supported by a network of brain regions, including the prefrontal cortex and hippocampus. For instance, the hippocampus encodes recurring patterns across different environments, establishing cognitive schemas that help macaques generalize knowledge and apply it to new situations (Gray and Barnes 2019). This cognitive flexibility enables macaques to adapt to new challenges and solve problems in real‐time, demonstrating the adaptive value of flexible representations.

### 2.4 Emerging Evidence in the Ferret

While much of the research on animal intelligence focuses on primates, other species, like ferrets, also offer valuable insights into the neural and behavioral mechanisms underlying cognitive abilities. In ferrets, intelligence manifests in their ability to solve problems essential for survival in their natural environment. This includes behaviors that go beyond simple reflexes, involving more complex problem‐solving skills. For instance, in laboratory experiments, ferrets are often tested on their ability to perceive and respond to visual stimuli, such as motion or contrast (Dunn‐Weiss et al. 2019). These tasks require the ferrets to understand that the visual stimulus contains information useful for solving the task, highlighting their capacity for learning and adaptation.

The initial training sessions in these experiments are particularly challenging for the ferrets, as they must first figure out the rules of the task. This process involves recognizing that the stimulus is relevant to the problem at hand and determining how to use this information to achieve the desired outcome. This ability to learn and adapt to new tasks underscores the ferrets’ problem‐solving skills, which are indicative of their intelligence. Although these tasks primarily test visual perception, the underlying cognitive processes are similar to those involved in more complex problem‐solving scenarios in natural environments.

Despite these capabilities, there may be certain limits to the intelligence of ferrets, largely related to their physical and anatomical characteristics. Ferrets do not use their paws for complex manipulative tasks like tool use (Vinke et al. 2021), which suggests a limitation in their ability to perform certain types of problem‐solving tasks that require dexterity. Additionally, it is assumed that ferrets may struggle with tasks involving self‐recognition and understanding others, such as recognizing themselves in a mirror or understanding the intentions of other ferrets. However, these assumptions have not been rigorously tested in experiments. While recent evidence indicates they may be aware of their physical dimensions (Khatov 2023), it remains an open question as to what extent ferrets can demonstrate these more complex cognitive abilities.

In cases where cognitive flexibility has been studied in ferrets, the focus has been on decision‐making based on sensory processing rather than social cognition or visuospatial cognitive maps. In laboratory experiments, ferrets must learn to recognize that visual stimuli contain information useful for solving tasks (Dunn‐Weiss et al. 2019). The initial training sessions, where they must figure out the task rules, highlight their capacity for learning and adaptation. This flexibility in sensory processing and neural plasticity allows ferrets to adjust their behavior based on changing environmental conditions, demonstrating a form of cognitive flexibility essential for their survival. While ferrets may not possess sophisticated cognitive maps based on social hierarchy as do macaques, their ability to adapt their behavioral strategies to new sensory experiences provides valuable insights into the neural mechanisms underlying flexible representations.

When considering intelligence across model systems, it is important to consider the ethological relevance of the tasks used to assess their cognitive abilities. Macaques and ferrets, like all animals, have evolved sets of core cognitive functions necessary for survival in their distinct natural environment. These functions include problem‐solving abilities that are directly related to their ecological niches, such as navigating their surroundings, finding food, and avoiding predators. In highly social species like macaques, this flexibility is evident in their sophisticated social cognition and spatial navigation abilities. In ferrets, flexibility is demonstrated through their capacity for sensory adaptation and learning in response to new stimuli. While ferrets may not possess the same level of intelligence as primates in terms of social cognition or tool use, their cognitive abilities are well‐suited to their specific environmental challenges. Overall, flexible representations are a key component of intelligence in both macaques and ferrets, enabling them to adapt to their respective environments. Understanding these flexible representations provides valuable insights into the cognitive processes that underlie complex behaviors and adaptations across different species, highlighting the diverse manifestations of intelligence in the animal kingdom.

## 3 Uncaging Intelligence: The Cognitive Capabilities of Crows

Crows, often considered one of the most intelligent bird species, exhibit a diverse array of behaviors that highlight their cognitive prowess. Notably, they display impressive problem‐solving abilities, such as using tools to extract food from hard‐to‐reach places or manipulating objects to create tools suited to their needs (von Bayern et al. 2018; Boeckle et al. 2020). Social learning is another remarkable trait observed in crows, where individuals learn from observing and interacting with conspecifics, leading to the transmission of knowledge across generations (Jelbert et al. 2018). Additionally, crows demonstrate complex communication skills, utilizing a variety of vocalizations and gestures to convey information within their social groups (Kondo 2012; Mates et al. 2015). Their ability to uniquely recognize individuals, both within their own species and other species, further emphasizes the sophistication and complexity of social representations in crows (Kondo et al. 2012; Brecht et al. 2017). Moreover, studies have documented instances of crows exhibiting sensory awareness (Nieder et al. 2020), partially evidenced by their ability to recognize themselves in mirrors (Buniyaadi et al. 2020; Prior et al. 2008). A trait previously thought to be exclusive to primates, this is now known to be found across more diverse taxa, though still in a minority of species. These observations align with more refined definitions of intelligence, emphasizing problem‐solving abilities and cognitive control functions as central aspects of intelligence.

### 3.1 Neural Bases: A Very Different Brain, With Very Similar Circuit Concepts

Despite possessing brains significantly smaller than those of mammals, crows have demonstrated cognitive abilities comparable to some primates, raising questions about the neural underpinnings of their intelligence. While the neuroanatomy of avian brains differs substantially from that of mammals, studies have revealed intriguing parallels in the organization of neural circuits involved in cognitive processes (Güntürkün et al. 2021). The telencephalon, particularly the pallium, plays a pivotal role in intelligence among vertebrates. Despite independently evolved anatomical structures in birds and mammals, the physiology of the telencephalon likely holds the key to intelligence in both groups.

The avian pallium is not layered as in mammals, but rather “nucleated” or “nodal,” where functional brain regions are grouped together in a densely packed locus, in contrast to the layered organization of the cerebral cortex (Jarvis et al. 2005). Despite this gross organizational difference, there exists evidence of laminar and columnar organization within the avian sensory pallium, implying functional columns of information processing similar to cortex (A. Wang et al. 2010; Dugas‐Ford et al. 2012; Stacho et al. 2020). While both birds and primates possess similar brain regions involved in basic functions such as sensory processing and motor control, they also have unique neuroanatomical structures. For example, birds have a structure called the Wulst, which is involved in visual processing and is analogous to the mammalian visual cortex (Nieder and Wagner 2000; Bischof et al. 2016; Stacho et al. 2020). Additionally, birds have a unique structure called the nidopallium, which is involved in higher‐order cognitive functions such as learning, memory, and problem‐solving (Rose and Colombo 2005; Rinnert et al. 2019).

The most conspicuous difference in avian brains is perhaps the generally smaller size of the avian brain, likely reflecting different ecological pressures and behavioral repertoires. Despite this small size, avian brains have one of the highest packing densities of neurons of any brain, with about two to four times as many neurons as rodent brains of similar size and about twice as many neurons as primate brains of similar size (Olkowicz et al. 2016). In addition, birds have remarkably high ratios of neurons to nonneuronal cells in the pallium, where neurons constitute approximately 60%–80% of cells (Olkowicz et al. 2016), in contrast to mammals, where only about 20% of cortical cells are neurons (Azevedo et al. 2009; Herculano‐Houzel et al. 2006; Herculano‐Houzel et al. 2007). These differences are striking, particularly when considering many birds—including various songbirds, parrots, and hummingbirds—exhibit vocal communication behaviors that are highly complex and learned, and can also be culturally transmitted, similar to the way humans learn language (Hahn et al. 2021). These peculiarities in avian brains highlight the potential for diverse and efficient processing of information that likely underlies intelligence.

**FIGURE 1:**
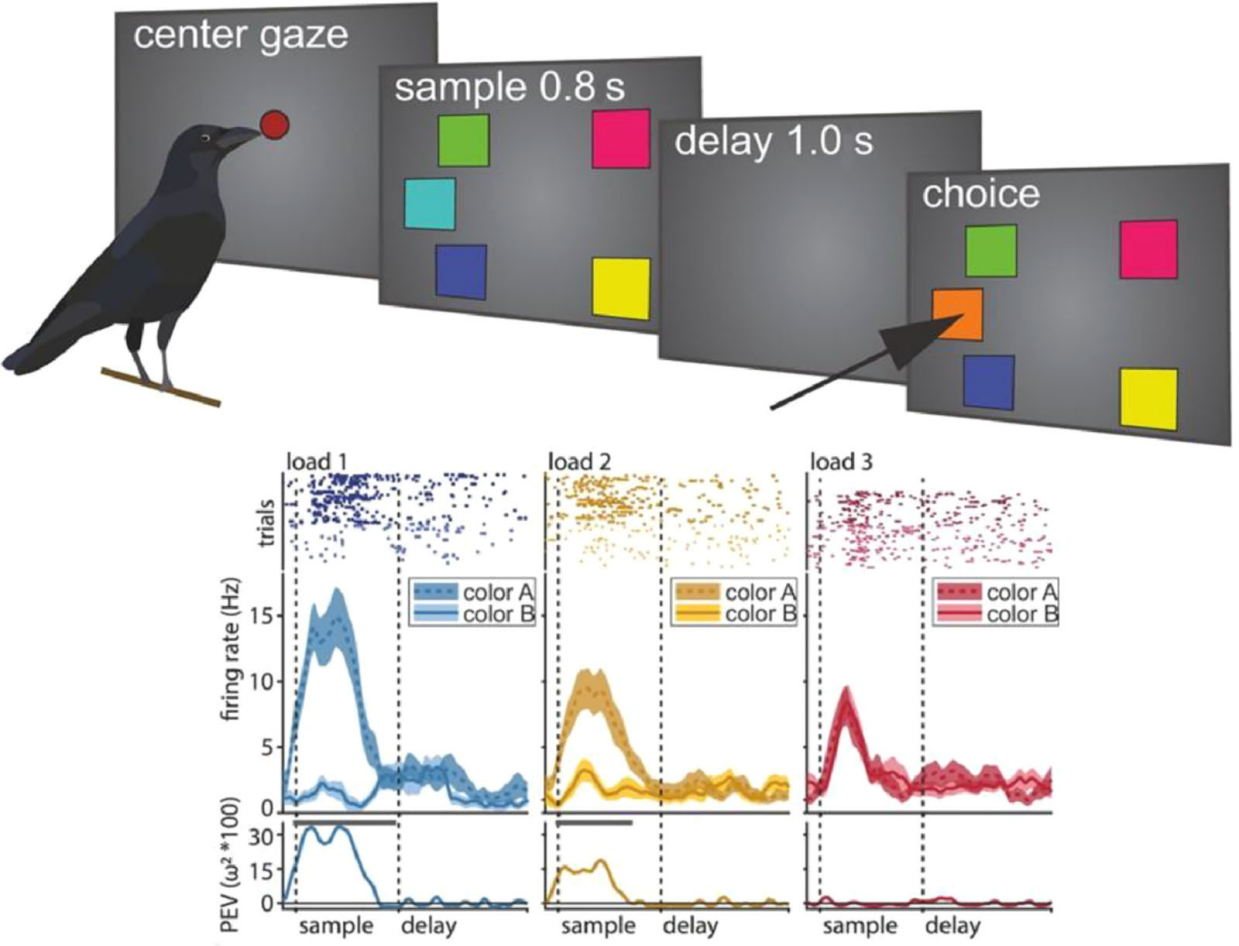
A working memory task in the crow and its neural correlates. Top: A working memory behavioral paradigm in the crow. The birds first have to center and maintain their gaze for the duration of the sample period and the delay period. Then, during the choice period, they must indicate which of the colored squares have changed, here marked with a black arrowhead. Bottom: Neural responses during this task for three different load conditions are shown. More activity during the sample period than during the delay period is shown at three different levels: raster plots, where each dot represents a single spike during the individual trials; peristimulus time histogram, or average firing rates; and percent explained variance of color identity. Adapted from Hahn et al. (2021).

Both crows and primates exhibit similar patterns of neuronal activity during tasks requiring working memory (Figure 1) (Bock 2024), suggesting convergent evolution of neural mechanisms underlying this cognitive function. Furthermore, recent advances in neuroimaging techniques have enabled researchers to map the neural circuits that may be involved in various cognitive tasks in crows (Marzluff et al. 2012; Pendergraft et al. 2023), using activity‐dependent fluorescent tracers that can then be imaged while the bird is under anesthesia, giving a proxy of which parts of the brain were active during these tasks. Studies such as these have revealed intriguing similarities with those observed in mammals. The discovery of recurrent excitation and sustained calcium/GluN2B activity for working memory in both crows and primates further underscores the conservation of essential circuit concepts across distantly related taxa (Arnsten et al. 2021; Herold et al. 2022; M. Wang et al. 2013; Ditz et al. 2022). Thus, despite differences in brain size, structure, and circuitry, crows appear to employ similar neural strategies to accomplish cognitive tasks, highlighting the remarkable evolutionary convergence of intelligence‐related neural circuitry.

### 3.2 “Algorithms of Intelligence” That Give Rise to Similar Cognitive Operations

The concept of “algorithms of intelligence” posits that certain neural mechanisms give rise to similar cognitive operations across species, offering insights into the general principles underlying intelligence. By elucidating these shared mechanisms, researchers can unravel the computational strategies employed by different species to solve cognitive tasks. For instance, comparative studies of working memory mechanisms in crows and primates have revealed striking similarities in the neural circuits involved, suggesting the existence of common algorithms for this cognitive function (Veit and Nieder 2013; Wagener and Nieder 2024; Liao et al. 2024; Carandini and Heeger 2012). Furthermore, investigations into the neural basis of problem‐solving and decision‐making in diverse taxa have uncovered conserved patterns of neuronal activity underlying these processes, indicative of shared computational principles (Carandini and Heeger 2012). The identification of recurrent excitation and sustained calcium/GluN2B activity as key components of working memory across distantly related species further supports the notion of universal algorithms of intelligence. Thus, by studying the concepts of neural mechanisms shared across species, researchers can gain valuable insights into the fundamental principles governing cognitive processes and intelligence evolution. This understanding of shared neural mechanisms can inform not only our understanding of animal cognition but also artificial intelligence and neuroengineering endeavors, highlighting the interdisciplinary significance of studying intelligence across species.

## 4 Eight is Enough: Deep Sea Intelligence in the Octopus

The subject of octopus intelligence has received considerable attention in popular culture, and anecdotally, the octopus is widely regarded as a highly intelligent creature. Octopuses are challenging to observe in their ecological niches, given their sophisticated camouflage capabilities and their ability to traverse great depths. In the lab, hyper‐reduced conditions, even with enrichment, may diminish the behavioral flexibility these creatures exhibit in the wild, and tasks designed for land vertebrates are difficult to translate. These challenges have historically impeded scientific inquiry into their intelligence and nervous system organization. We have now begun to use cutting‐edge molecular tools to develop single‐cell taxonomies and utilize genetic manipulations (Baden et al. 2023), to explore circuit organization using serial electron microscopy (Bidel et al. 2023), observe correlates of complex cognition (Mather and Dickel 2017), and even speculate about their level of consciousness (Ponte et al. 2022). In another significant advance by Gutnick, Neef, et al. (2023), we now have a paradigm to record neural activity in the brain of the freely moving octopus, a laudable accomplishment given the soft, boneless, flexible body of an octopus and their ability to tamper with attached equipment.

**FIGURE 2:**
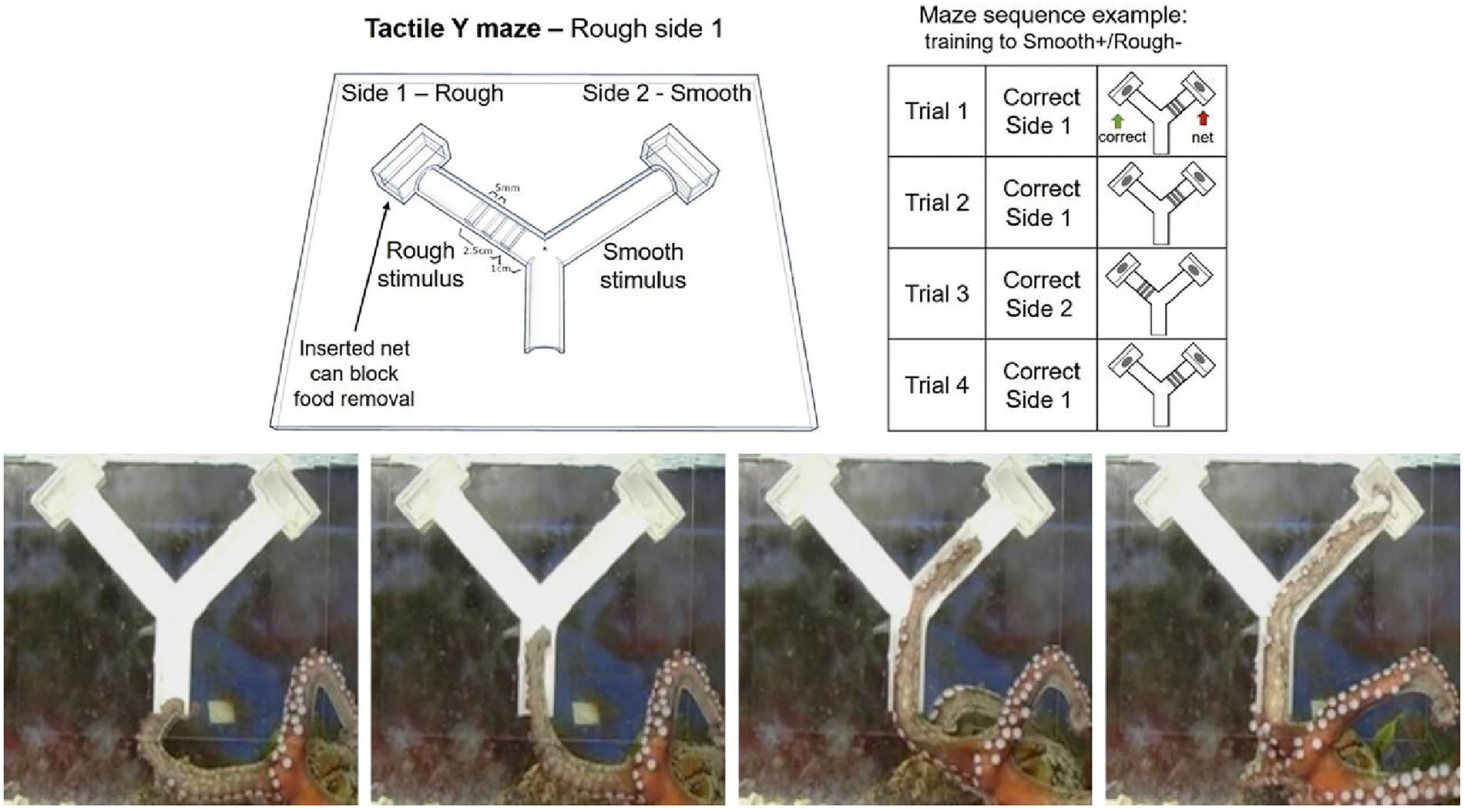
A behavioral task in the octopus. Top: An example of a Y‐maze construction for the octopus, designed for using tactile information. Side 1 has a rough stimulus, and Side 2 has a smooth stimulus. In this example, the octopus must learn to associate the tactile stimulus in the entry arm with a food reward at the end of one of the arms. Bottom: Octopuses were trained to insert their arms down the left or right side of the maze based on tactile stimulus in the entry arms. Here, the octopus correctly identifies the tactile stimulus in the right arm of the maze and retrieves the food reward. Adapted from Gutnick et al. (2022).

One of the first challenges in investigating octopus intelligence is simply to understand how sensory sampling is integrated to form perception. Octopus brains have variable sensory modules depending on their ecological niche. Benthic octopuses, which locomote out of the water and onto land, have more brain volume dedicated to tactile and visual processing than pelagic species that remain submerged (Gutnick and Rokhsar et al. 2023), suggesting that ecological niche powerfully influences the evolutionary forces shaping brain anatomy and organization (Ponte et al. 2021; Gutnick et al. 2022). But all octopuses have roughly 2/3 of their neurons situated outside of the central nervous system, distributed among peripheral and axial nervous systems that course through their eight “arms,” which are substantially devoted to chemosensation and somatosensation and possibly even aspects of vision (Figure 2) (Gutnick et al. 2022). Even so, cephalopods have the highest degree of brain centralization among invertebrates, including analogs to the thalamus and cortex, among other key vertebrate structures (Ponte et al. 2022; Shigeno et al. 2018), suggesting that similar organizational motifs might have emerged independently across taxa to support intelligence‐like behaviors.

Beyond sensory integration, another important question is how attention is allocated, and decisions are made. We do not understand if the octopus attends to a single or selection of arms at a time, and if so, how the hierarchy is established. Studying octopus motor behavior presents a unique opportunity for neuroscientists to understand how such a divergent system accomplishes common tasks, such as using an arm to grasp for a visually identified target (Gutnick et al. 2011; Bidel et al. 2022; Zullo et al. 2019). Because of its soft, flexible arms with infinite degrees of freedom, it could theoretically use any arm to do any task, but emerging evidence suggests that arms can be dedicated to specific tasks (Bidel et al. 2022). Arms are often characterized as independent because amputated arms can perform comparable movements as intact arms, suggesting limited feedback from a central source (Zullo et al. 2019). Yet, studies suggest that movements in *Octopus vulgaris* might be represented within higher motor centers (called basal lobes) by overlapping and redundant circuits that may not be somatotopically organized as they are in most vertebrates (Zullo et al. 2019; Hochner et al. 2023; Zullo et al. 2009; Zullo and Hochner 2011).

Octopuses demonstrate what might be considerable behavioral flexibility and adaptability and have a smaller repertoire of stereotyped behaviors and fixed action patterns than other invertebrates (Ponte et al. 2022). For example, *Octopus vulgaris* has been observed shifting to a nocturnal hunting schedule to avoid a specific lurking diurnal predator, but seemingly not bothering to change its hunting schedule when other predators that hunt both diurnally and nocturnally are nearby (Gutnick et al. 2022). Octopuses are also known to utilize a variety of objects for shelter and protection (Ponte et al. 2021), and even during hunting. Pliny the Elder, in the first century ad, anecdotally reported watching an octopus wait for a large Mediterranean clam to open its shell in order to prop it open with a stone, as discussed by Ponte and colleagues (Ponte et al. 2022). Mather and Dickel (Mather and Dickel 2017) present an array of evidence for complex cognition in cephalopods. Individual octopuses show variability in their approaches to solving experimental tasks, sometimes referred to as “personalities” (Borrelli et al. 2020; Ponte et al. 2022; Gutnick, Rokhsar, et al. 2023).

While much of the historical work in octopus behavioral studies has been anecdotal, often falling short of the experimental standards used for vertebrates, more recent work by Gutnick, highlighted above, and others, have aimed to translate behavioral paradigms from other species for more rigorous interrogation in the octopus and other cephalopods. It is obvious that there is much work to be done in setting up the infrastructure for meaningful and reproducible behavioral assessment across a wider range of modalities. However, it is an essential endeavor toward understanding the neural capabilities of these impressive creatures, and highlights a greater need to focus on species diversity to better formalize general concepts of intelligence. While octopuses do fail at experimental tasks (Gutnick et al. 2011; Dews 1959), one simple reason may be that we as experimenters are not yet asking them “the right questions” ‐ meaning that we are failing to coregister our intelligence‐measuring heuristics, which are highly anthropomorphized, to the motivational and perceptual space appropriate to a creature so dissimilar to us. In summary, we, as experimenters and observers, may have to adapt our own assumptions and approaches to adequately understand these inscrutable animals.

## 5 Winged Wisdom: The Emerging Neural Bases of Insect Intelligence

Bees exhibit a range of remarkable behaviors that are underpinned by their relatively simple neural architecture. Bees are known for their complex foraging behaviors (Brunet et al. 2023), sophisticated navigation abilities (Patel et al. 2024), and intricate social structures (Michener 1974; Bloch and Grozinger 2011). These behaviors are supported by neural circuits that, while simpler than those of vertebrates, are highly specialized and efficient (Watanabe et al. 2017; Stone et al. 2017). For instance, honey bees (*Apis mellifera)* can engage in complex tasks that require learning and memory, such as associating specific flowers with rewards (Muth et al. 2016), demonstrating a notable level of cognitive flexibility and problem‐solving ability. According to Giurfa (Giurfa 2021), honey bees are adept at learning sameness and difference relationships, a type of conceptual rule learning based on physical features of stimuli, which signifies a deeper cognitive processing capability that allows them to navigate their ecological surroundings efficiently.

The navigation abilities of bees are especially remarkable. They utilize a combination of visual landmarks and the sun's position to navigate (Kheradmand and Nieh 2019), and their brains contain dedicated regions for processing these navigational cues (Paulk et al. 2008; Mertes et al. 2014). This capability not only enhances their foraging efficiency but also reflects an advanced level of spatial awareness. The neural circuits involved in these tasks demonstrate how a relatively small brain can sustain complex cognitive processes, suggesting that, similar to birds mentioned above, intelligence is not solely dependent on brain size but rather on the intricacies and organization of neural circuits.

Bumblebees (Bombus spp.), in particular, have lately garnered attention for their social learning skills, which were showcased in recent research by Bridges and colleagues (Bridges et al. 2024). These studies revealed that bumblebees can learn complex behaviors through social observation, acquiring skills that are too difficult for them to innovate independently. This highlights the potential for culture‐like behaviors and cultural transmission in insect societies. This suggests that intelligence in bees is not merely an individual trait but can be socially transmitted, adding further nuance to our understanding of cognitive ecology and animal intelligence.

Flies, particularly the fruit fly *Drosophila melanogaster*, have been extensively studied for their neural and behavioral capabilities. Despite their small brain size, flies exhibit complex behaviors such as courtship rituals (Pavlou and Goodwin 2013), predator evasion (Muijres et al. 2014; Parigi et al. 2019), and efficient foraging strategies (Whitehead et al. 2024). The central complex in the fly brain is involved in navigational computations and motor coordination, allowing flies to adapt their flight patterns and foraging behaviors based on environmental cues (Fisher 2022). While flies may not display the same level of individual intelligence as some other species, their neural circuits provide valuable insights into the basic mechanisms of sensory processing, decision‐making, and motor control.

The intelligence exhibited by octopuses, bees, and flies is closely tied to their ecological niches and survival strategies. Octopuses, with their sophisticated camouflage and problem‐solving abilities, are well‐adapted to diverse and challenging marine environments. Bees’ navigational skills and social behaviors ensure efficient foraging and colony survival. Flies’ rapid sensory processing and motor coordination allow them to thrive in a variety of habitats, from fruit orchards to urban environments. These examples highlight how intelligence, in its various forms, is shaped by evolutionary pressures and ecological demands, leading to specialized adaptations that enhance survival and reproductive success. Understanding these cognitive traits in invertebrates not only enriches our knowledge of insect intelligence but also provides valuable insights into the broader field of neuroscience—especially concerning how intelligence can emerge from relatively simple neural circuits.

## 6 Climate Control: Navigating a Changing World

One topic the NSF posed as an emerging question in neuroscience broadly is the relationship between intelligence and climate change—in other words, do intelligent behaviors help a species deal with climate change and survive on a warming planet on relatively short time scales? This question is particularly salient, given projections for how incremental increases in global temperatures born out of climate change directly impact the increasing extinction risk of the planet's species (Parmesan et al. 2022). Evolutionary pressures and ecological demands that have shaped adaptations across species are long‐term processes, typically only observable over long time scales. Behaviors are often quantified in a more narrow view—e.g., in experimentally controlled tasks, in a laboratory setting, and in ways that may or may not have direct ethological relevance to a real‐world environment. Yet, flexibility or adaptability of behaviors, a central aspect of intelligence, is likely to be highly relevant to dealing with changing environments, globally and locally, on both long and short time scales.

Flexibility is crucial for a species to adapt to rapidly changing environments. Intelligent behaviors that demonstrate adaptability enable organisms to respond to new challenges posed by climate change. Flexibility in behavior allows animals to modify their strategies in response to environmental changes, such as shifts in temperature, availability of resources, or the introduction of new predators. This adaptability can mean the difference between survival and extinction.

For instance, while migratory patterns in birds are largely innate and influenced by genetic selection (Pulido 2007), many species can exhibit secondary behaviors in response to local environmental conditions—such as altering their feeding strategies or nesting sites in reaction to changes in temperature and food availability (Plummer et al. 2015; Londe et al. 2021). Another pertinent example of intelligent behavior is found in species capable of using tools or innovating based on environmental challenges. New Caledonian crows have shown remarkable problem‐solving skills, enabling them to adapt to varying food sources or predatory threats (Rutz and St Claire 2012). Octopuses also exhibit remarkable behavioral adaptability, utilizing visual information to guide their independent arms, which allows for complex movements in response to varied environmental stimuli (Gutnick et al. 2011). This ability to integrate sensory information and coordinate complex movements demonstrates a high degree of neural sophistication and behavioral adaptability. More broadly, these behaviors demonstrate a degree of cognitive flexibility and learning that is vital for survival in a changing world and illustrate behavioral adaptability stemming from cognitive capabilities rather than purely genetic predispositions.

Species with longer lifespans, such as humans, require greater flexibility to survive over extended periods (Jones 2011). These species need reliable long‐term memory to retain knowledge about past environmental conditions and to predict and plan for future challenges (Klein et al. 2010). Species that demonstrate higher cognitive abilities often have more extended life histories, enabling them to develop strategies that enhance their survival over time. This relationship suggests that intelligence may not be merely a trait for immediate problem‐solving but a fundamental aspect of a species' adaptation and resilience in the face of environmental changes (Dunkel et al. 2021). Skeptical evaluation of information and the ability to plan ahead are crucial for making informed decisions that ensure long‐term survival. Since longer‐lived species must continually adapt to changing environments, this necessitates a high degree of behavioral flexibility, a hallmark of intelligence.

The cognitive buffer hypothesis offers a compelling explanation for how intelligence enhances survival. This hypothesis posits that a larger brain fosters behavioral flexibility, which allows individuals to cope more effectively with environmental variability. Such cognitive capabilities can lead to innovative problem‐solving, which is essential for navigating dynamic ecosystems (Sol et al. 2016, Sol et al. 2009). For instance, species exhibiting complex foraging behaviors show a better capacity to adjust to resource availability and environmental shifts, thereby reducing their extinction risk. Evidence suggests that birds able to innovate—by using new tools or foraging techniques—are less likely to face extinction in changing habitats (Ducatez et al. 2020; Audet et al. 2024).

Research has indeed shown that larger relative brain sizes in various animal species are often associated with increased behavioral flexibility. For example, studies have indicated that successful individuals in invasive species tend to have larger brains than their unsuccessful counterparts, enabling them to better respond to novel challenges in new environments (Amiel et al. 2011; Szabo et al. 2020). Other work also indicates that, while animals with larger brains are better able to adapt to variable environments, it was the evolution of larger brains may have been an essential development for these abilities (Fristoe et al. 2017). These trends underscore the possible significant role of cognitive abilities in facilitating adaptability. Yet, as already discussed, brain size might not be the best proxy of cognitive capabilities, and there may be other important cellular contributors to intelligence, such as differences in neuron packing density, circuit differences, developmental trajectories, and neuron/nonneuronal ratios between the different species (Rinnert et al. 2019; Olkowicz et al. 2016; Azevedo et al. 2009; Herculano‐Houzel et al. 2006; G. A. Wildenberg et al. 2021; G. Wildenberg et al. 2023). Thus, while brain size might be a useful, if gross, preliminary metric when considering flexibility across closely related species, it may fail in describing behavioral flexibility across more distant clades occupying more dissimilar ecological niches.

Flexibility is especially important when dealing with significant environmental changes, such as those brought about by climate change. While innate behaviors play a critical role in all species, the ability to learn and adjust behaviors, such as foraging strategies or habitat use in response to climatic shifts, can greatly enhance survival. Animals that can modify their behaviors in response to increasing climatic variability or food scarcity—which might be universally found in adaptable species—are, therefore, better equipped to thrive in the face of challenges posed by climate change (Ducatez et al. 2020; Audet et al. 2024). As environments become more unpredictable, the ability to quickly adapt to new conditions becomes a critical survival trait.

In conclusion, behaviors that promote flexibility are vital for species facing the challenges of climate change. Whether through individual adaptability or the collective diversity of cognitive abilities within a species, flexibility enhances resilience and increases the chances of survival in an ever‐changing environment. As we continue to study the neural bases and behavioral repertoires of various species, we gain a deeper understanding of how intelligence and adaptability might be intertwined, offering insights that may help us address our own challenges with climate change.

## 7 Final Intelligent Thoughts

Here, we considered intelligence as the measure of an agent's ability to achieve goals in a wide range of environments. The intelligence exhibited across the animal kingdom challenges traditional notions of vertebrate cognition. Intelligence should not be a species‐specific definition and should rather be broader across animals, with many taxa exhibiting remarkable intelligence. Macaques exhibit impressive and flexible navigation and working memory capabilities, perhaps mirroring their similar neural circuit organizational motifs as compared with humans. Their use of these capabilities in the domains of social cognition and future planning is particularly stunning. Abstract thinking in birds is equally outstanding, driven out of similar circuit concepts as mammals. Insects such as bees and flies exhibit a wide range of flexible, intelligent behaviors, particularly in abstract rule learning and social transmission of knowledge. By studying the neural bases and behavioral repertoire of diverse species, we gain valuable insights into the universality of intelligence across species. These inform not only our understanding of animal cognition but also artificial intelligence, neuroengineering endeavors, and, ultimately, the neural bases of intelligent behavior.

Despite anatomical differences—in brain size, neuronal density, and circuit architecture—shared neural mechanisms underlie cognitive processes in diverse species. Macaques and ferrets have very different brain sizes in terms of mass, yet exhibit similar problem‐solving abilities that seem to be supported by similar types of neurons and circuits. Birds, despite their small brain sizes relative to mammals, have some of the highest neuronal densities in their analog of the cortex, often two to four times more than mammals with brains of similar size. Their differences in gross anatomical organization of the brain do not extend to circuit concepts, where laminar and columnar organization similar to mammals is observed in the sensory pallium of birds, and recurrent excitation and related functional circuit concepts underlie working memory processes, as in mammals. Relatively small and simple neural organizational motifs can also give rise to incredibly complex behaviors, such as impressive navigational and social cognition behaviors in bees and flies. Intelligence can even arise through a more distributed neural organization, such as in octopuses, where the majority of neurons are located outside of the central brain. It is clear that intelligent behaviors are widespread across the animal kingdom despite incredible gaps between tangible brain measures and intelligence, an issue compounded by the incredible diversity in neural circuits across species. While much work has focused on brain size as a descriptor for the capability for intelligence, this may have value primarily when comparing within individuals of a species or across highly similar species. It is obvious that these rules, and others equating brain measures to intelligence, might fall apart when comparing across more distantly related taxa. Thus, in order to more equivocally define intelligence—both in behaviors and in the neural circuits from which they arise—a return to more comparative work is essential for answering these and other outstanding questions.

Significant disparities likely exist in the spectrum of intelligence amongst animal species, highlighting the need for a broader comparative approach in our studies. Such an approach would undoubtedly advance our understanding of the diversity of intelligence across species. More importantly, it would aid in deciphering the evolution of intelligence, including what ecological pressures, genetic predispositions, and neural circuit concepts might give rise to intelligent behaviors. While all animals are adapted to their environments and demonstrate impressive survival and reproductive abilities, these traits do not necessarily equate with intelligence.

Ethological relevance and innate sources of adaptability inform our understanding of an animal's behavioral repertoire, and thus the ways in which such intelligence might be observed. Yet, this should not be equated with intelligence. Intelligence encompasses problem‐solving abilities and cognitive control functions, enabling adaptive decision‐making in dynamic environments. Thus, an animal's adaptability to changing environments might be a testament to its possible intelligence, just as an animal that excels solely in a constrained realm such as foraging might not be considered intelligent. Intelligence should transcend species‐specific definitions, and it should not be domain‐specific but domain‐general. Thus, we gain more insight into domain‐general definitions of intelligence by making broader inquiries across domains and species.

Does defining intelligence in animal model systems help neuroscience? It is a difficult question, rooted in a complex phenomenon. While we cannot say for certain, we argue that the pursuit is essential to understanding the brain and certainly merits more intelligent thought from the community. A return to a more comparative approach in our work is integral to more meaningfully advance our understanding of complex phenomena such as intelligence, disentangling its biological and evolutionary roots, and broadening our conception of the rules that help define it. As we continue to unravel the mysteries of the brain, perhaps our greatest lesson will be that intelligence, in all its forms, is as varied and adaptable as life itself.

## Author Contributions

Conceptual design: Joseph V. Gogola, Mary Kate Joyce, Susheel Vijayraghavan, George Barnum, and Gregg Wildenberg. Drafted the manuscript: Joseph V. Gogola. Critical revisions: Joseph V. Gogola, Mary Kate Joyce, Susheel Vijayraghavan, George Barnum, and Gregg Wildenberg. Final approval: Joseph V. Gogola, Mary Kate Joyce, Susheel Vijayraghavan, George Barnum, and Gregg Wildenberg. Supervision: Gregg Wildenberg.

### Peer Review

The peer review history for this article is available at https://publons.com/publon/10.1002/cne.70035.

## Data Availability

The authors have nothing to report.
